# Optimizing Health Coaching for Patients With Type 2 Diabetes Using Machine Learning: Model Development and Validation Study

**DOI:** 10.2196/37838

**Published:** 2022-09-13

**Authors:** Shuang Di, Jeremy Petch, Hertzel C Gerstein, Ruoqing Zhu, Diana Sherifali

**Affiliations:** 1 Centre for Data Science and Digital Health Hamilton Health Sciences Hamilton, ON Canada; 2 Dalla Lana School of Public Health University of Toronto Toronto, ON Canada; 3 Population Health Research Institute Hamilton Health Sciences Hamilton, ON Canada; 4 Institute of Health Policy, Management and Evaluation University of Toronto Toronto, ON Canada; 5 Department of Medicine Faculty of Health Sciences McMaster University Hamilton, ON Canada; 6 Department of Statistics University of Illinois at Urbana-Champaign Champaign, IL United States; 7 School of Nursing McMaster University Hamilton, ON Canada

**Keywords:** diabetes health coaching, artificial intelligence, reinforcement learning, health coaching, patient outcome, diabetes, community health, digital intervention, health outcome

## Abstract

**Background:**

Health coaching is an emerging intervention that has been shown to improve clinical and patient-relevant outcomes for type 2 diabetes. Advances in artificial intelligence may provide an avenue for developing a more personalized, adaptive, and cost-effective approach to diabetes health coaching.

**Objective:**

We aim to apply Q-learning, a widely used reinforcement learning algorithm, to a diabetes health-coaching data set to develop a model for recommending an optimal coaching intervention at each decision point that is tailored to a patient’s accumulated history.

**Methods:**

In this pilot study, we fit a two-stage reinforcement learning model on 177 patients from the intervention arm of a community-based randomized controlled trial conducted in Canada. The policy produced by the reinforcement learning model can recommend a coaching intervention at each decision point that is tailored to a patient’s accumulated history and is expected to maximize the composite clinical outcome of hemoglobin A_1c_ reduction and quality of life improvement (normalized to [ ​0, 1 ​], with a higher score being better). Our data, models, and source code are publicly available.

**Results:**

Among the 177 patients, the coaching intervention recommended by our policy mirrored the observed diabetes health coach’s interventions in 17.5% (n=31) of the patients in stage 1 and 14.1% (n=25) of the patients in stage 2. Where there was agreement in both stages, the average cumulative composite outcome (0.839, 95% CI 0.460-1.220) was better than those for whom the optimal policy agreed with the diabetes health coach in only one stage (0.791, 95% CI 0.747-0.836) or differed in both stages (0.755, 95% CI 0.728-0.781). Additionally, the average cumulative composite outcome predicted for the policy’s recommendations was significantly better than that of the observed diabetes health coach’s recommendations (*t*_n-1_=10.040; *P*<.001).

**Conclusions:**

Applying reinforcement learning to diabetes health coaching could allow for both the automation of health coaching and an improvement in health outcomes produced by this type of intervention.

## Introduction

Chronic diseases are a major health care challenge globally and domestically, being the leading cause of death and disability worldwide as of 2021 [1,2] and accounting for 89% of all deaths in Canada [3]. As of 2011, type 2 diabetes (T2D) affects more than 2.5 million people in Canada specifically and costs the health care system over CAD $6.7 billion (US $5.1 billion) to treat annually [4].

Health coaching is quickly emerging as a new approach to partner with patients to optimize their self-management through lifestyle changes [5]. Diabetes health coaching has both educational and behavioral components, which include goal-setting, self-care knowledge, and frequent follow-up appointments [6]. Coaching has been shown to improve health outcomes [7-9] and treatment adherence [10,11]. However, the widespread adoption of diabetes health coaching may be limited by constraints on health human resources. Artificial intelligence incorporated into a digital health platform could automate some routine health-coaching tasks to improve the scalability of coaching interventions. Moreover, artificial intelligence may be able to leverage data from a patient’s history that is not routinely used in clinical practice to optimize coaching recommendations.

**Figure 1:**
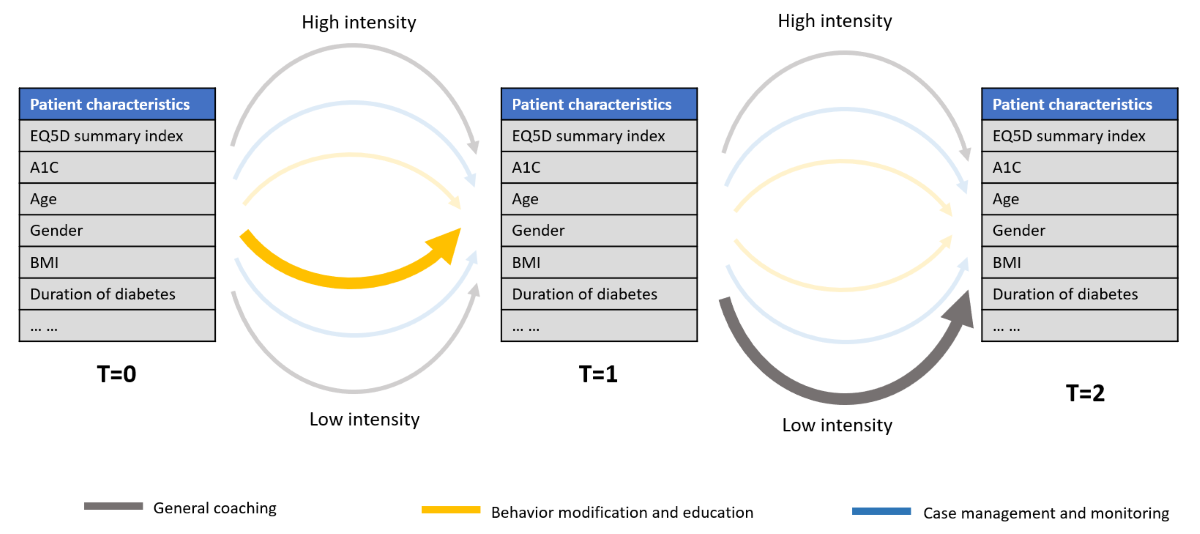
Diabetes health-coaching optimization as a sequential decision-making problem. A_1c_: Hemoglobin A_1c_; ED5D: EuroQol five-dimension scale questionnaire; T: time step.

Recent work in artificial intelligence and medicine [12] suggests that individual patient data can be leveraged to assist the decision-making process of diabetes health coaching and suggests incremental adjustments of interventions tailored to the patient’s changing needs and health status. Reinforcement learning is commonly used for estimating an optimal set of actions (called a “policy”) for this type of sequential decision-making problem [13,14]. Reinforcement learning works by iteratively choosing actions and, then in turn, is rewarded based on the outcomes of those actions. This is done for every set of patient characteristics at every time step in a data set. The algorithm “learns” the best action to take at every time step by maximizing the value of the rewards over all time steps for each patient (Figure 1).

Several studies have applied reinforcement learning for diabetes management, with most focused on controlling blood glucose levels [15,16]. Vrabie et al [17] used reinforcement learning to obtain optimal adaptive control algorithms for dynamical systems using mathematical models. Ngo et al [18,19] applied reinforcement learning for the optimal control of blood glucose in patients with type 1 diabetes and proposed a reinforcement learning algorithm for automatically calculating the basal and bolus insulin doses for patients with diabetes using a simulation on a blood glucose model.

Relatively few studies have used reinforcement learning for diabetes health coaching [20-22]. Yom-Tov et al [20] developed a reinforcement learning–powered system that used personalized messages to improve T2D patients’ compliance with their physical activity regimens. Lauffenburger et al [22] have developed a reinforcement learning–powered system to personalize SMS text messages to promote medication adherence. This existing reinforcement learning research for diabetes health coaching focuses on improving coaching within a single domain (only physical activities or only medication adherence). Our study is the first to use reinforcement learning to recommend comprehensive coaching strategies that can include all domains of coaching (physical activity, medication adherence, diet modification, etc) that are tailored based on patients’ changing clinical status and ongoing performance.

In this study, we applied Q-learning (Multimedia Appendix 1), a widely used reinforcement learning algorithm, to a diabetes health-coaching data set to develop a model for recommending an optimal coaching intervention at each decision point that is tailored to a patient’s accumulated history.

## Methods

### Data Overview

The data set used in this study was collected in a community-based randomized controlled trial conducted in Ontario, Canada [23]. Patients in the trial were 18 years or older, diagnosed with T2D (any duration), and had a hemoglobin A_1c_ (HbA_1c_) level >7.5% within 6 months prior to randomization. All patients were able to read and write in English, and had access to a telephone. Those excluded were pregnant women, had debilitating coexisting conditions (ie, mental illness or impaired cognition), or had underlying medical conditions that may have provided misleading HbA_1c_ levels. A total of 365 patients were randomized using a 1:1 ratio into the intervention (diabetes health coaching) or control (usual care) groups. All patients in the intervention arm of the trial were included in the current analysis.

Patients in the intervention arm received both standard care and an additional diabetes health-coaching intervention. Standard care included receiving access to usual diabetes education (individual or group) provided by nurses or dietitians, typically every 3 to 6 months, along with community resources. In addition, the intervention group received diabetes health coaching delivered by a registered nurse or certified diabetes educator that emphasized small positive habits customized to one’s environment, ability, and motivation. The topic or agenda of each telephone call was determined by the participant or was agreed upon in the previous coaching session. All patients in the intervention arm had access to diabetes health coaching for 1 year.

For each patient, the data set contained demographic data, including age, gender, ethnicity, diabetes duration, and comorbidities; clinical characteristics, including BMI, weight, and most recent HbA_1c_; health care resource use information, including hospital admissions, emergency room visits, specialist visits, and other health care visits (eg, nurse visits); and quality of life (QoL) measures. Demographic data and health care resource use were collected using self-reported questionnaires, and clinical characteristics were assessed at study visits or through medical records. QoL was measured using three scales, including the Audit of Diabetes-Dependent Quality of Life (ADDQoL) scale [24], the Diabetes Self-Care Activities (DSCA) scale [25], and the EQ-5D scale [26]. All the measures were collected at baseline and at the 6-month and 12-month follow-ups. A coaching intervention use form was used to document the diabetes health coaching received by each patient over the course of the trial. A patient could visit the diabetes health coach multiple times during the trial and could receive one or multiple coaching recommendations at each visit: dietary modification, exercise modification, behavioral modification, medication adherence, medication adjustment, glucose monitoring, psychological support or counseling, case management/monitoring, and system navigation.

We have made the data set used in this study publicly available [27].

### Ethical Approval

The trial from which our data set was derived was approved by the Hamilton Integrated Research Ethics Board (approval/file number: 14-416). Written informed consent was obtained from all participants (inability to provide informed consent was an exclusion criteria for the trial) and included permission for a secondary analysis without additional consent. The trial was registered at ClinicalTrials.gov (NCT02128815) [28]. All data for the trial was deidentified. Participants were provided a small honorarium of CAD $20 (US $15.25) per visit for over three visits (thus, a total of CAD $60 [US $45.75]) for participation in the trial.

### Problem Formulation for the Reinforcement Learning Model

Reinforcement learning is an approach to machine learning inspired by how animals and humans can learn new tasks through receiving rewards for desirable behavior. For example, dogs are often taught to perform tricks by giving them treats after performing well. In reinforcement learning, an algorithm (referred to as an “agent”) learns an optimal policy through trial and error within a simulated environment. During the learning process, the agent will make decisions based on inputs from the environment and then will receive rewards if those decisions resulted in a desirable outcome. Over many iterations, the agent eventually learns an optimal strategy (referred to as a “policy”) that allows it to consistently maximize rewards.

In this study, our goal was to use reinforcement learning to learn an optimal policy for recommending diabetes coaching interventions at each clinical decision point, using a patient’s accumulated history as inputs. We rewarded the agent based on a composite outcome of HbA_1c_ reduction and QoL improvement (measured using the EQ-5D summary index, which was chosen based on expert clinical input). We set both weights to 0.5 to reflect equal importance and additionally scaled both HbA_1c_ reduction and QoL improvements to the range of [​0, 1​] before calculating the weighted average. For example, 1 patient had an HbA_1c_ of 7.0 and an EQ-5D summary index of 0.457 at baseline, and then at the 6-month follow-up, their HbA_1c_ decreased to 6.8 and their EQ-5D summary index increased to 0.533. We calculated their reduction in HbA_1c_ as 0.835 (2.86% reduction before min-max scaling) and their increase in QoL as 0.504 (16.5% improvement before min-max scaling). The weighted average was calculated as 0.5 * 0.835 + 0.5 * 0.504, which is 0.670. The reinforcement learning agent was rewarded based on the cumulative composite outcome, which we calculated by adding together the composite outcome as recorded at both the 6-month and 12-month follow-ups.

To prepare the simulated environment necessary for reinforcement learning, we first identified the patient characteristics, decision points, and intervention options from the data set. Patient characteristics included demographic data, clinical characteristics, health care resource use information, and self-reported QoL measured by the ADDQoL and DSCA scales. Since we had access to patient characteristics and the outcome of interest measured at baseline, the 6 month follow-up, and the 12-month follow-up, we formalized the sequence of data as a 2-stage estimation problem, with the 2 decision points being the initial visit and the 6-month follow-up.

In reinforcement learning, the available options for interventions are called the “action space.” Action spaces that are too complex can cause challenges with the learning process, so it is standard practice in reinforcement learning to “shape” the action space by constraining the available options in some way—often by combining very similar actions [29]. For our study, we grouped 9 distinct diabetes coaching recommendations into 3 categories based on expert clinical input. Specifically, dietary modification, exercise modification, and behavioral modification were grouped into the category of behavior modification and education; medication adherence, medication adjustment, glucose monitoring, case management/monitoring, and system navigation were grouped into the category of case management and monitoring; and psychological support and counseling were combined into the category of psychological support. To be classified as one of these 3 categories, a patient needed to have at least twice as many recommendations in 1 category compared to the others—otherwise they were classified as a fourth category: general coaching.

**Figure 2:**
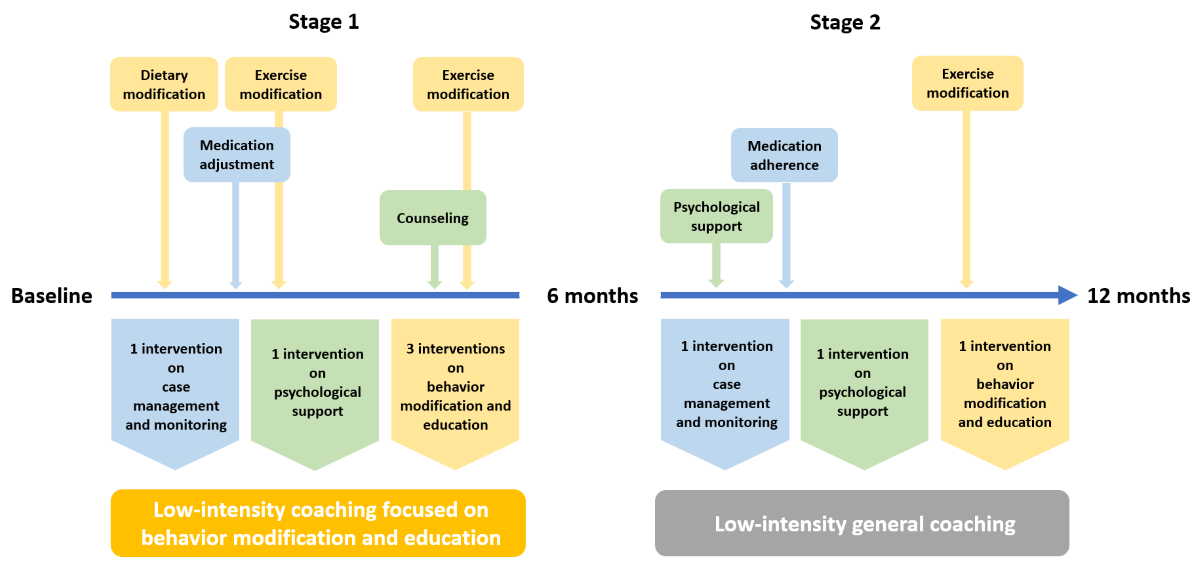
An example of a single patient in the data set.

In addition to shaping the action space based on the focus of the interventions, we also categorized interventions based on intensity. We categorized intensity by calculating the total number of coaching recommendations received by each patient in a stage to obtain the median of the total number of coaching recommendations among all patients. High-intensity coaching was categorized as being greater than the median number of coaching recommendations during a time interval, and low-intensity coaching was categorized as fewer than the median. The dimensions of focus and intensity resulted in an action space with 8 possible actions (Figure 2).

### Optimal Policy Estimation and Validation

Following problem formulation, we fit a reinforcement learning model to “learn” which interventions tend to produce the best outcome for each set of patient characteristics. The Q-learning algorithm formulates this as a prediction problem, with patient characteristics and coaching actions as model inputs used to predict the cumulative composite outcome, which we use as the reward function. This prediction model is then used to select the optimal action for a given set of patient characteristics by estimating the rewards for all possible actions from the action space and selecting the one estimated to produce the greatest reward. While any regression modeling technique can be used for this type of prediction problem, we selected histogram-based gradient boosting classification trees [30], as they are better suited to modeling large numbers of patient characteristics with complex interactions than techniques like linear regression.

Given the relatively small sample size available for this pilot, we were able to use a leave-one-out cross-validation (LOOCV) approach [31] for model development and validation. This approach trains a model on all available patients but 1, then uses the remaining patient for model validation. This process is then repeated for every possible split of the data, resulting in 177 iterations of train/test for our data set. This hypothesis was tested using a paired *t* test.

The potential clinical effectiveness of our model was evaluated using two approaches. The first approach compared the model predicted cumulative composite outcome with the actual observed outcome (the observed result of the interventions provided by the diabetes health coaches). We hypothesized that our model’s predicted outcome would be higher than the observed outcome.

The second approach assessed the relationship between the cumulative composite outcome and the proportion of agreement between our model and the diabetes health coach. We hypothesized that higher levels of agreement between our model and coach recommendations would be associated with better observed outcomes and that lower levels of agreement would be associated with worse outcomes.

The level of agreement between the reinforcement learning model and the observed diabetes health coach’s interventions was not used to evaluate the performance of our model. This is because reinforcement learning assumes that there is room for improvement over observed behavior. Thus the goal is to learn a different better policy than what was observed, rather than simply mirroring what was done by the coaches.

We have made the source code and trained models developed for this study publicly available [27].

## Results

A total of 177 patients in the intervention arm of the community-based randomized controlled trial were included in the analysis. Distributions of the patient characteristics used as model inputs at baseline and the 6-month and 12-month follow-ups are summarized in Table 1. *P* values are reported to illustrate the degree of change for each characteristic between time points.

The median of the total number of coaching recommendations received in stage 1 and stage 2 was 8. Following the prespecified criteria for defining intervention options, we obtained the following intervention options (Table 2).

Among the 177 patients, LOOCV results showed that the average cumulative composite outcome expected by the reinforcement learning model (0.811) was significantly higher than the observed outcome (0.767; *t*_n-1_=10.040; *P*<.001).

LOOCV results also showed that our model mirrored the observed diabetes health coach’s interventions in 17.5% (n=31) of the patients in stage 1 and in 14.1% (n=25) of the patients in stage 2. Among the patients for whom our model agreed with the diabetes health coach in both stages, the average cumulative composite outcome (0.839, 95% CI 0.460-1.220) was better than those for whom our model agreed with the diabetes health coach in only one stage (0.791, 95% CI 0.747-0.836) or differed in both stages (0.755, 95% CI 0.728-0.781).

**Table 1:** Patient characteristics used as model inputs with SDs and percentages at baseline, the 6-month follow-up, and the 12-month follow-up (N=177).

Patient characteristics		Baseline	6-month follow-up	12-month follow-up	*P* value	
Age (years), mean (SD)		57.4 (11.3)	57.4 (11.3)	57.4 (11.3)	N/A^a^	
**Gender, n (%)**						N/A
	Female	94 (53.1)	94 (53.1)	94 (53.1)		
	Male	83 (46.9)	83 (46.9)	83 (46.9)		
**Ethnicity, n (%)**						N/A
	Caucasian	141 (79.7)	141 (79.7)	141 (79.7)		
	Latin American	10 (5.6)	10 (5.6)	10 (5.6)		
	South Asian	10 (5.6)	10 (5.6)	10 (5.6)		
	Aboriginal	3 (1.7)	3 (1.7)	3 (1.7)		
	Filipino	3 (1.7)	3 (1.7)	3 (1.7)		
	Black	2 (1.1)	2 (1.1)	2 (1.1)		
	Southeast Asian	2 (1.1)	2 (1.1)	2 (1.1)		
	Arab	1 (0.6)	1 (0.6)	1 (0.6)		
	Chinese	1 (0.6)	1 (0.6)	1 (0.6)		
	West Asian	1 (0.6)	1 (0.6)	1 (0.6)		
	Unknown	3 (1.7)	3 (1.7)	3 (1.7)		
BMI, mean (SD)		34.5 (6.9)	34.1 (7.2)	33.6 (6.9)	.51	
Duration of diabetes (years), mean (SD)		9.4 (9.1)	9.9 (9.1)	10.4 (9.1)	.60	
Hemoglobin A_1c_, mean (SD)		9.1 (1.7)	7.6 (1.2)	7.3 (1.1)	<.001	
Family physician visits, mean (SD)		3.0 (2.9)	2.4 (2.3)	2.2 (1.9)	.003	
Family physician visits related to diabetes, mean (SD)		1.7 (1.6)	1.6 (1.6)	1.4 (0.9)	.11	
Visits with health professional, n (%)		45 (25.4)	55 (31.1)	80 (45.2)	<.001	
Emergency room and hospital admissions, n (%)		156 (88.1)	160 (90.4)	116 (65.5)	<.001	
Chronic disease management program, n (%)		164 (92.7)	173 (97.7)	126 (71.2)	<.001	
**Behavioral stage, n (%)**						<.001
	Action	103 (58.2)	128 (72.3)	104 (58.8)		
	Contemplation	15 (8.5)	6 (3.4)	3 (1.7)		
	I am not sure	2 (1.1)	1 (0.6)	0 (0.0)		
	Maintenance	0 (0.0)	24 (13.6)	52 (29.4)		
	Precontemplation	7 (4.0)	0 (0.0)	0 (0.0)		
	Preparation	50 (28.2)	18 (10.2)	18 (10.2)		
**Diabetes treatment, n (%)**						
	Diet	85 (48.0)	70 (39.5)	43 (24.3)	<.001	
	Oral therapy	163 (92.1)	166 (93.8)	165 (93.2)	.82	
	Insulin	70 (39.5)	77 (43.5)	76 (42.9)	.72	
	Other	3 (1.7)	2 (1.1)	0 (0.0)	.24	
EQ-5D summary index, mean (SD)		0.8 (0.2)	0.8 (0.1)	0.8 (0.1)	.06	
ADDQoL^b^ summary score, mean (SD)		–1.5 (1.3)	–1.4 (1.1)	–1.3 (0.7)	.10	
**Diabetes Self-Care Activities scale**						
	General diet, mean (SD)	4.5 (2.8)	5.6 (2.3)	6.1 (1.9)	<.001	
	Specific diet, mean (SD)	5.1 (1.6)	5.4 (1.3)	5.6 (1.1)	.003	
	Exercise, mean (SD)	4.1 (2.6)	5.3 (2.4)	5.6 (2.3)	<.001	
	Blood glucose testing, mean (SD)	5.3 (2.4)	5.8 (2.0)	5.5 (2.2)	.13	
	Foot care, mean (SD)	2.9 (1.9)	3.5 (1.3)	2.9 (1.7)	<.001	
	Current smoker, n (%)	29 (16.4)	24 (13.6)	23 (13.0)	.62	
	Cigarettes smoked per day, mean (SD)	2.8 (7.2)	2.3 (7.2)	2.0 (6.8)	.62	
	Additional diet^c^, mean (SD)	3.7 (3.2)	4.9 (3.1)	5.3 (2.9)	<.001	
	Additional medication, mean (SD)	6.7 (1.4)	6.9 (0.9)	6.9 (0.9)	.18	
	Additional foot care, mean (SD)	5.9 (1.4)	6.4 (1.1)	6.5 (0.8)	<.001	
Stroke, n (%)		5 (2.8)	0 (0.0)	1 (0.6)	.03	
Transient ischemic attack, n (%)		17 (9.6)	1 (0.6)	0 (0.0)	<.001	
Evidence of coronary artery disease, n (%)		17 (9.6)	0 (0.0)	0 (0.0)	<.001	
Myocardial infarction, n (%)		4 (2.3)	0 (0.0)	0 (0.0)	.02	
Heart failure, n (%)		0 (0.0)	0 (0.0)	1 (0.6)	.37	
Kidney disease, n (%)		10 (5.6)	1 (0.6)	0 (0.0)	<.001	
Chronic obstructive pulmonary disease, n (%)		19 (10.7)	3 (1.7)	3 (1.7)	<.001	
Hyperlipidemia, n (%)		94 (53.1)	6 (3.4)	0 (0.0)	<.001	
Hypertension, n (%)		108 (61.0)	9 (5.1)	0 (0.0)	<.001	
Peripheral arterial disease, n (%)		3 (1.7)	2 (1.1)	0 (0.0)	.24	
Prescribed medications, n (%)		5 (2.8)	45 (25.4)	29 (16.4)	<.001	

^a^N/A: not applicable.

^b^ADDQoL: Audit of Diabetes-Dependent Quality of Life.

^c^Additional items for the expanded version of the summary of Diabetes Self-Care Activities.

**Table 2:** Intervention options.

Intervention	Coaching recommendations (stage 1), n	Coaching recommendations (stage 2), n
High-intensity general coaching	45	18
High-intensity coaching on case management and monitoring	34	18
High-intensity coaching on behavior modification and education	29	10
Low-intensity general coaching	23	64
Low-intensity coaching on case management and monitoring	30	42
Low-intensity coaching on behavior modification and education	16	25

## Discussion

The study took a novel approach of developing artificial intelligence using diabetes health-coaching data to better fit the needs of diabetes management and to achieve better health outcomes. Using historical observational data from a community-based randomized controlled trial, we developed a reinforcement learning model that can automate the task of personalized adaptive diabetes health coaching and demonstrates the potential to outperform human diabetes health coaches in maximizing a composite outcome of HbA_1c_ reduction and QoL improvement. Our approach is also able to leverage data that is often overlooked, such as self-reported behavioral data, which allows us to generate personalized adaptive interventions for each patient using comprehensive health data.

The model-based decision-making process is fully automated, which requires less involvement from health care professional resources. In practice, our model could be integrated into existing diabetes health-coaching programs to dynamically suggest personalized adaptive coaching interventions, either as a decision-making support tool for the diabetes health coaches or combined with a patient-facing mobile app to directly support patients with diabetes, which has the potential to reduce the cost and expand the reach of diabetes health coaching [32,33].

This study has several limitations. The internal working of the reinforcement learning model is difficult to interpret, and as a result, the model appears as a black box to health care professionals and patients, which may present a barrier to adoption in some clinical settings [34]. Due to the relatively small sample size, the data source for this study lacks heterogeneity, which may result in insufficient generalizability of the estimated optimal policy, despite its satisfactory performance on the study population. We plan to address this limitation in future work, which will seek to include a larger and more diverse group of patients. The aggregation of detailed diabetes health-coaching data into discrete intervention options may have led to a loss of fidelity, which in turn may translate into less optimal intervention recommendations. Future work in this area may look to more advanced statistical methods to fully use the fine-grained original coaching information to produce a better performance. Finally, diabetes health coach’s interventions can potentially have different consequences on patients due to the human factors (eg, patients’ adherence to coaching) that cannot be fully simulated, which may lead to lower performance in real-world clinical practice. Future work should investigate quantifying these human factors and including them in the reinforcement learning model.

This pilot study presents a novel application of artificial intelligence in diabetes management and demonstrated that applying reinforcement learning to diabetes health-coaching data has the potential to automate coaching and yield substantial improvement in health outcomes. Future research will include applying the reinforcement learning approach to larger diabetes health-coaching data sets and exploring the feasibility and acceptability of diabetes health coaching supported by artificial intelligence.

## Appendix

Multimedia Appendix 1 Q-learning.

## Abbreviations

ADDQoL: Audit of Diabetes-Dependent Quality of Life
DSCA: Diabetes Self-Care Activities
HbA_1c_: hemoglobin A_1c_
LOOCV: leave-one-out cross-validation
QoL: quality of life
T2D: type 2 diabetes
