# Supplementary material for: Optimizing Health Coaching for Patients With Type 2 Diabetes Using Machine Learning: Model Development and Validation Study
Source: JMIR Form Res. 2022 Sep 13;6(9):e37838. doi: 10.2196/37838 (PMC9516374; doi:10.2196/37838)
Supplement: Multimedia Appendix 1 [file formative_v6i9e37838_app1.docx]

**Multimedia Appendix 1**

**Q-learning**

Q-learning can be viewed as an extension of regression to sequential decision-making problems. A simple two-stage scenario can be formalised using the following elements:

1. $o_{k}$: tailoring variables available for decision-making.
2. $a_{k}:$intervention.
3. $h_{k}$: accumulated history, including all tailoring variables and interventions preceding $a_{k}$.
4. $y_{k}$: outcome.

* Note: k ∈ {1, 2} indexes the first and second treatment stages.

The Q-function at stage 2 measures the expected cumulative outcome of assigning $a_{2}$ to a patient presenting with history $h_{2}$, while the Q-function at stage 1 measures the expected cumulative outcome of assigning $a_{1}$ to a patient presenting with history $h_{1}$, assuming an optimal decision rule will be followed at stage two. The conditional expectations that define the Q-functions are approximated with regression models and the Q-learning algorithm involves the following backward induction procedure:

1. Estimate the stage 2 Q-function by regressing $y_{2}$ on $a_{2}$ and $h_{2}$, and recommend an intervention $a_{2}^{*}$ that maximizes $\hat{y_{2}}$.
2. Compute stage 1 pseudo-outcome as $y_{1}+\hat{y_{2}}$.
3. Estimate the stage 1 Q-function by regressing $y_{1}+\hat{y_{2}}$ on $a_{1}$ and $h_{1}$, and recommend an intervention $a_{1}^{*}$ that maximizes $y_{1}+\hat{y_{2}}$.

This procedure allows to learn an optimal policy using observational data generated following a suboptimal policy and can be easily generalized to multiple-stage problems.

**Model details**

The iterative Q-learning approach used in thus study trains a gradient boosting regression tree (GBRT) model to predict the reward at each timestep. We optimize the hyperparameters of the GBRT model (learning rate, number of iterations, and L2 regularization) by grid search, using expected reward as the objective.
